# *TIMP3* and *CCNA1* hypermethylation in HNSCC is associated with an increased incidence of second primary tumors

**DOI:** 10.1186/1479-5876-11-316

**Published:** 2013-12-20

**Authors:** Marianna Marconato Rettori, Ana Carolina de Carvalho, Ana Luiza Bomfim Longo, Cleyton Zanardo de Oliveira, Luiz Paulo Kowalski, André Lopes Carvalho, André Luiz Vettore

**Affiliations:** 1 Cancer Molecular Biology Laboratory, Department of Biological Sciences, Federal University of São Paulo, 04039-020, São Paulo, Brazil; 2 Statistics and Epidemiology Center, Barretos Cancer Hospital, 14784-400, Barretos, Brazil; 3 Departament of Head and Neck Surgery, A.C. Camargo Hospital, 01509-010, São Paulo, Brazil; 4 Departament of Head and Neck Surgery, Barretos Cancer Hospital, 14784-400, Barretos, Brazil; 5 Cancer and Stem Cell Biology Program, Duke-NUS Graduate Medical School, 169857, Singapore, Singapore

**Keywords:** Head and neck cancers, HNSCC Prognostic Marker, DNA methylation, epigenetics, *TIMP3*, *CCNA1*

## Abstract

**Background:**

Hypermethylation in the promoter regions is associated with the suppression of gene expression and has been considered a potential molecular marker for several tumor types, including head and neck squamous cell carcinomas (HNSCC).

**Methods:**

To evaluate the gene hypermethylation profile as a prognostic marker, this retrospective study used a QMSP approach to determine the methylation status of 19 genes in 70 HNSCC patients.

**Results:**

The methylation profile analysis of primary HNSCC revealed that genes *CCNA1, DAPK, MGMT, TIMP3* and *SFRP1* were frequently hypermethylated, with high specificity and sensitivity. *TIMP3* and *CCNA1* hypermethylation was significantly associated with lower rates of second primary tumor-free survival (p = 0.007 and p = 0.001; log-rank test, respectively).

**Conclusion:**

This study, for the first time, presents *CCNA1* and *TIMP3* hypermethylation as a helpful tool to identify HNSCC subjects at risk of developing second primary carcinomas.

## Background

Head and neck squamous cell carcinomas (HNSCC) are the sixth most common non-skin cancer in the world, with an incidence of 600,000 cases per year [1]. Despite improvements in diagnosis and management of HNSCC patients, through combined efforts in prevention, surgery, radiotherapy and chemotherapy, a significant percentage of patients still have a poor prognosis with a five-year survival of only 50% [2].

High recurrence and second primary tumor (SPT) rates are common reasons for HNSCC treatment failure [3,4]. With an incidence of 17-30% and an annual risk of 3-10% [4-6], the development of SPT significantly contributes to a worse prognosis and cancer-associated death for HNSCC patients [4,7-10]. The SPT development is in accordance with the field cancerization theory, which refers to the presence of malignant or premalignant changes in the entire apparently normal mucosa in response to carcinogen exposition, especially tobacco and alcohol [11].

Some potential molecular markers have been evaluated aiming to identify genetic abnormalities associated with a possible prediction of SPT [12-14]. Aberrant DNA methylation (hypermethylation) of gene promoter region acts as an alternative to mutations in disrupting tumor suppressor gene function [15]. This process involves the addition of a methyl group to the carbon 5 position of the cytosine ring in CpG dinucleotides catalyzed by DNA methyltransferases [16]. It is associated with several changes in chromatin structure and the recruitment of proteins to the methylated sites. The methylation usually leads to the obstruction of the promoter region, hindering gene transcription and subsequently causing gene silencing [17]. Genes involved in the cell cycle control, DNA repair, apoptosis, cell adhesion and signal transduction have already been described as inactivated by aberrant promoter methylation in different human cancers [18,19] including HNSCC [20,21]. DNA hypermethylation can be measured in tissue samples or body fluids using a real-time quantitative methylation-specific PCR (QMSP) approach. The ability to quantify methylation allows the delineation of clinically meaningful threshold values of methylation to improve sensitivity and specificity in the detection of tumor-specific signal [16,22,23].

We have previously reported that evaluation of methylation profile in salivary rinses is as an independent prognostic marker for local recurrence-free survival in patients with HNSCC, justifying the use of DNA hypermethylation detection in saliva as a tool for identifying and monitoring HNSCC patients’ subgroups with high risk of presenting local recurrence [24].

Patients who develop an SPT have a significantly worse prognosis and increased risk of death by cancer. Thus, the best strategies to improve patient management are prevention, early diagnosis, an appropriate treatment choice and close follow-up of patients, with deep investigation of all suspicious lesions. The feasibility of using molecular markers able to predict the outcome of HNSCC, through the evaluation of gene methylation patterns in samples from HNSCC patients, largely opens the potential for a better therapy choice and closer surveillance after treatment of the primary tumor. Thus, in this retrospective study, we sought to characterize the promoter methylation status of 19 genes in primary tumors from HNSCC patients, and evaluate its clinical significance and usefulness as a prognostic biomarker, especially regarding the prediction of the development of second primary tumors in HNSCC patients.

## Methods

### Patients

This retrospective study involved tissue specimens from 70 HNSCC patients who underwent tumor resection between 2006 and 2010 at the Department of Head and Neck Surgery of the A. C. Camargo Hospital (São Paulo, Brazil). These samples were available at the tumor bank of the A. C. Camargo Hospital. Only patients diagnosed with primary HNSCC, not previously treated, that were over 18 years of age, treated with curative intent and presenting with tumors at oral cavity, larynx, or pharynx were included in the study. All samples were checked microscopically for the presence of neoplastic tissue and the absence of contaminating normal mucosa. Tissue samples were snap-frozen in liquid nitrogen within 30 minutes after resection and stored at -80°C. For the control group, 60 salivary rinse samples from healthy accompanying patients (68% men, median age 46.3 and 38% smokers) were collected at the Barretos Cancer Hospital (São Paulo, Brazil).

The experimental protocol was approved by the Ethics Committees of the A. C. Camargo Hospital and performed in accordance with the ethical guidelines of the 1975 Declaration of Helsinki. Clinical-pathological information was collected from the patients’ medical records. Smoking was defined as use of tobacco, chewable or smoked, for at least 1 year continuously. Alcohol use was defined as intake of more than 2 alcoholic drinks per day, for at least 1 year continuously.

### Sample collection and DNA extraction

Genomic DNA was isolated from the tissue samples using the TRIzol reagent (Invitrogen, Frederick, MD) following manufacturer’s recommendations. Salivary rinses were obtained by swishing and gargling with 10 mL normal saline solution (NaCl 0.9%). Samples were centrifuged for 10 minutes at 1,500 rpm, cell pellets were suspended in 300 μL of water and stored at -70°C. DNA from exfoliated cells present in salivary rinse was extracted by digestion with 50 mg/mL proteinase K (Invitrogen) in the presence of 1% SDS at 48°C overnight, followed by phenol/chloroform extraction and ethanol precipitation.

### Bisulfite treatment

Bisulfite treatment of DNA converts unmethylated cytosines to uracil, while the methylated ones remain as cytosines. Sodium-bisulfite conversion of 2 μg of DNA was performed according a previously described method with modifications [25]. In brief, 2 μg of DNA from each sample was denatured in 0.2 M NaOH for 20 min at 50°C (in a total volume of 20 μL). The denatured DNA was diluted in 500 μL of a freshly made bisulfite solution (2.5 M sodium metabisulfite, 125 mM hydroquinone, 350 mM sodium chloride, pH5.0) and incubated for 3 h at 70°C in the dark. Bisulfite-modified DNA was purified using the Wizard DNA Clean-Up System (Promega) according to the manufacturer’s instructions and eluted in 45 μL of water at 80°C. After treatment with NaOH (final concentration 0.3 M) for 10 min at room temperature, the treated DNA was precipitated by the addition of 75 μL of ammonium acetate, 2.5 volumes of ethanol, and 2 μL of glycogen (5 mg/mL). Each resulting DNA pellet was washed with 70% ethanol, dried, dissolved in 110 μL of water, and stored at -80°C.

### Target gene selection

A total of 19 genes were selected for the examination of methylation abnormalities. The panel included genes reported as targets for epigenetic silencing in different human cancers. All the genes evaluated in this study present tumor suppressor activities and their silencing could contribute to the tumorigenesis process. Among these genes are *CCNA1, CCND2, CDKN2B, DAPK, DCC, COX2* and *SOCS1* which are involved in cell cycle control and apoptosis, *CDH1, THBS1* and *TIMP3* in cell adhesion, *RARβ* and *TGFβR2* in signal transduction processes, *MGMT* in DNA repair, *CALCA* and *MT1G* in cell-cell signaling processes, *HIC1, SFRP1, UCHL1* and *HIN1* in cell differentiation and proliferation. It has been shown that the expression of these genes may be affected by aberrant promoter methylation in association with transcription silencing in different types of human malignancies [20,26-31].

### Quantitative methylation-specific PCR

The quantitative methylation-specific PCR analyses (QMSP) were conducted as previously described [32]. Basically, 30 ng of bisulfite-modified DNA (this is the amount of material enough to ensure that a methylated sample will be detected, given the sensitivity thresholds of the technique*)* was used as template in fluorogenic QMSP assays carried out in a final volume of 20 μL in 96-well plates in the ABI Prism SDS 7500 (Applied Biosystems). PCR was performed in separate wells for each primer/probe set and each sample was run in triplicate. The final reaction mixture contained 3 μL of bisulfite-modified DNA, 1.2 μmol/L of forward and reverse primers, 200 nmol/L of the probe, 0.5U of platinum Taq polymerase (Invitrogen), 200 μmol/L dNTPs, 16.6 nmol/L ammonium sulfate, 67 mmol/L Trizma, 6.7 mmol/L magnesium chloride (2.5 mmol/L for *CDKN2A*), 10 mmol/L mercaptoethanol, 0.1% DMSO, and 1X ROX dye (Invitrogen). PCR was conducted with the following conditions: 95°C for 2 min, followed by 45 cycles at 95°C for 15 sec. and 60°C for 1 min. Each plate included patient DNA samples, multiple water blanks and serial dilutions (30–0.0003 ng) of a positive control allowing the construction of calibration curves. Leukocyte DNA obtained from a healthy individual was methylated *in vitro* using SssI methyltransferase (New England Biolabs) to generate methylated DNA at all CpG to be used as positive control.

Primers and probes were obtained from the literature and specifically amplify the promoter regions of the 19 genes of interest and the internal control gene, *ACTB*. Primer and probe sequences are provided in Additional file 1: Table S1. The relative DNA methylation level of the 19 genes in each sample was determined as a ratio of methylation specific PCR-amplified gene to *ACTB* and then multiplied by 100 for easier tabulation (average value of triplicates of gene of interest divided by the average value of triplicates of *ACTB* x 100; reactions presenting standard deviation of the triplicates greater than 0.5 was repeated). A cut-off value of ≥0.1% was used to score the samples as positive ones for the genes *CCNA1, MGMT* and *SFRP1,* while for *DAPK* and *TIMP3*, no cut-off values were used, since these genes were not methylated at all in the saliva samples evaluated from controls. Cut-off values were used to optimize sensitivity and specificity levels to better distinguish HNSCC patients from healthy individuals and to exclude very low-level background readings that can occur in certain individual for certain genes [18].

### Statistical analysis

Statistical analysis was performed using the software SPSS 19.0 for Windows. Categorical variables were compared using Pearson’s Chi-square test or Fisher’s exact test, as appropriate. Survival curves were calculated by Kaplan-Meier method and differences between groups were compared using the log-rank test. Second primary tumors were defined according to the criteria proposed by Warren and Gates [33]. The second primary tumor control time was defined as the interval between the date of initial treatment and the diagnosis of second primary tumor, while the overall survival interval was defined as the interval between the date of initial treatment and the last follow up visit/information or death. For all analysis we considered statistical significance when p-value < 0.05.

## Results

### Patient characteristics and clinical predictors

Seventy HNSCC patients were included in this study (Table 1). They were mainly male (80%), with ages ranging from 20 to 90 (median 59 years). Tobacco use or alcohol consumption (current or past) were found in 87.1% and 82.9%, respectively. Primary tumor sites included: oral cavity (52.9%), larynx (30%), oropharynx (11.4%), and hypopharynx (5.7%). Clinical tumor stage at diagnosis was cT1/cT2 in 38.6% of the cases and cT3/cT4 in 61.4% of the cases, and 58.6% of patients presented a clinically positive lymph node.

**Table 1:** Demographic and clinical characteristics of HNSCC patients included in the study (n = 70)

**Characteristic**		**N**	**%**
	Median, range	59.2-90	
Age	> 60 y.o.	38	54.3
	≤ 60 y.o.	32	45.7
Gender	Male	56	80.0
	Female	14	20.0
Tobacco	Yes	61	87.1
Consumption	No	9	12.9
Alcohol	Yes	58	82.9
Consumption	No	12	17.1
Tumor	Oral cavity	37	52.9
Site	Larynx	21	30.0
	Oropharynx	8	11.4
	Hypopharynx	4	5.7
cT Stage	cT1/cT2	27	38.6
	cT3/cT4	43	61.4
cN Stage	cN0	29	41.4
	cN+	41	58.6
First curative	Surgery	13	18.6
treatment	Radiotherapy	3	4.3
	Surgery + Radio	34	48.6
	Surgery + Radio + Chemo	9	12.9
	Radio + Chemo	11	15.7
Second primary	Yes	7	10.0
Tumor	No	63	90.0
Recurrence	Yes	32	45.7
	No	38	54.3

Surgery followed by radiotherapy was the treatment approach in 48.6% of the patients. The median follow up period for these patients was 29.2 months (range: 1 – 62.6 months). Recurrences occurred in 32 cases (45.7%) and 7 (10%) patients developed second primary tumors (SPT) in the upper aerodigestive tract (lung, tongue, esophagus and lip).

### Quantitative methylation-specific PCR in HNSCC samples

Due to the scarcity of DNA quantity after bisulfite treatment of many samples and the number of genes selected, it would be virtually impossible to evaluate all possible candidate genes in all samples. So, we firstly decided to conduct an exploratory study, and then a more limited set of “best” genes would be used in an expanded cohort of samples. The first step was to verify the hypermethylation status of 19 genes in salivary rinse samples collected from healthy individuals (controls, n = 20). Although tumor and salivary rinse are not identical tissues, we used this method because formal biopsy of the 60 noncancer patients was not logistically feasible and other studies have already shown that saliva is a reliable source of normal mucosa cells [20]. This analysis showed that *TGFβR2, CALCA*, *HIC1*, *SOCS1, RARβ, COX2*, *CDH1*, *THBS1*, *HIN1*, *CDKN2B*, *UCHL1*, *CCND2*, *MT1G* and *DCC* were frequently methylated in control samples, showing low specificity (Table 2). Therefore, these 14 genes were excluded from the study. The methylation pattern of the remaining 7 genes, identified as unmethylated in control samples, was profiled in 20 HNSCC specimens. This analysis revealed that hypermethylation of *CCNA1*, *DAPK*, *MGMT*, *SFRP1* and *TIMP3* was frequent in head and neck tumor (40-70%). So, these 5 genes that could better distinguish HNSCC tumors from control samples were selected to be tested in the expanded cohort of HNSCC specimens (n = 70) and control subjects (n = 60).

**Table 2:** Comparison of hypermethylation detection on HNSCC tumor specimens and normal control salivary rinse samples

**Genes**	**Control**		**HNSCC**		**Specificity% (95% CI)**	**Senstivity% (95% CI)**
	**Cases (n)**	**M n (%)**	**Cases (n)**	**M n (%)**		
*CCNA1*	60	2 (3)	67	22 (33)	97 (93–101)	33 (21–45)
*DAPK*	39	1 (3)	68	35 (51)	97 (93–101)	51 (38–64)
*MGMT*	57	2 (4)	67	14 (21)	96 (91–101)	21 (11–31)
*SFRP1*	20	0 (0)	58	36 (62)	100	62 (50–74)
*TIMP3*	60	2 (3)	70	37 (53)	97 (93–101)	53 (40–66)

M – Methylated.

**Figure 1:**
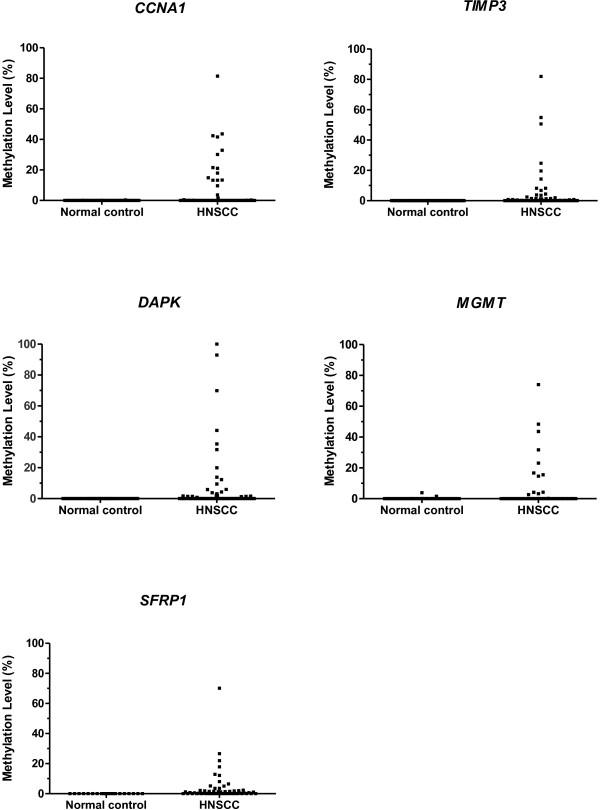
**The methylation rates of five genes (CCNA1, TIMP3, DAPK, MGMT and SFRP1) in normal controls and HNSCC samples.** x-axis, proportion of methylated cases/tested cases; y-axis, quantity of hypermethylation (gene of interest/ACTB × 100).

By the end, *CCNA1* was found methylated in 33% of HNSCC cases, *DAPK* in 51%, *MGMT* in 21%, *SFRP1* in 62% and *TIMP3* in 53% (Table 2, Figure 1). Noteworthy, complete coverage of every sample for every possible methylation marker selected was not possible due to either low quantity of total extracted DNA or limited DNA amount after bisulfite treatment. So, all the genes could not be run on all samples because of lack of DNA. This analysis demonstrated these genes as able to distinguish HNSCC tumors from control samples with high specificity (>96%) and sensitivity (21-62%) (Table 2). Furthermore, 54 HNSCC samples (77.1%) showed hypermethylation in at least one of these five genes.

### Association between aberrant methylation and patient characteristics

The methylation pattern of *CCNA1, DAPK, MGMT, SFRP1* and *TIMP3* as well as a panel containing all these 5 genes was analyzed for potential associations with clinical and pathological characteristics of HNSCC patients, including age, gender, tobacco consumption, alcohol consumption, primary tumor site, T stage, N stage, lymph vascular invasion, perineural invasion, surgical margins status, lymph node involvement and second primary tumor development.

This analysis showed that the hypermethylation of *CCNA1* (p = 0.007) and *SFRP1* (p = 0.024) was associated with age greater than 60 years old, while the hypermethylation of *TIMP3* was associated with hypopharynx tumors (p = 0.023; Table 3). Furthermore, aberrant methylation of *CCNA1* and *TIMP3* was significantly correlated to the development of SPT (p = 0.004 and p = 0.012, respectively). Of the 7 patients who developed SPT, 86% (6/7) had *CCNA1* methylated, while 100% showed *TIMP3* methylation (Table 3). There was no other significant association between gene hypermethylation and clinical and pathological characteristics of HNSCC patients.

**Table 3:** **Correlation between clinical and pathological characteristics of HNSCC patients and aberrant methylation profile of *CCNA1, DAPK, MGMT, SFRP1*, *TIMP3 *** **and the 5-gene panel in HNSCC samples**

**Characteristics**	**Category**	***CCNA1***		**p-value**	***DAPK***		**p-value**	***SFRP1***		**p-value**	***TIMP3***		**p-value**	**5-gene**	**panel**	**p-value**
		**U**	**M**		**U**	**M**		**U**	**M**		**U**	**M**		**U**	**M**	
**Age**	**< 60 yrs**	30 (81)	7 (19)	**0.007**	20 (53)	18 (48)	0.446	17 (50)	17 (50)	**0.024**	22 (56)	17 (44)	0.081	13 (34.2)	25 (65.8)	**0.016**
	**> 60 yrs**	15 (50)	15 (50)		13 (43)	17 (57)		5 (21)	19 (79)		11 (36)	20 (64)		3 (9.7)	28 (90.3)	
**Tumor site**	**Oral cavity**	26 (74)	9 (26)	0.530^a^	20 (56)	16 (44)	0.609^a^	15 (50)	15 (50)	0.055^a^	23 (62)	14 (38)	**0.023**^**a**^	9 (25.0)	27 (75.0)	0.419
	**Larynx**	13 (62)	8 (39)		8 (38)	13 (62)		5 (29)	12 (71)		7 (33)	14 (64)		6 (28.6)	15 (71.4)	
	**Oropharynx**	4 (57)	3 (43)		3 (43)	4 (57)		0 (0)	7 (100)		1 (13)	7 (87)		0 (0)	8 (100)	
	**Hypopharynx**	2 (50)	2 (50)		2 (50)	2 (50)		2 (50)	2 (50)		2 (50)	2 (50)		1 (25)	3 (75)	
**Second primary tumor**	**Absent**	44 (73)	16 (27)	**0.004**^**a**^	31 (51)	30 (49)	0.429^a^	21 (41)	30 (59)	0.235^a^	33 (52)	30 (48)	**0.012**^**a**^	16 (25.8)	46 (74.2)	0.188^a^
	**Present**	1 (14)	6 (86)		2 (29)	5 (71)		1 (14)	6 (86)		0 (0)	7 (100)		0 (0)	7 (100)	

U – unmethylated, n (%); M – methylated, n (%), ^a^ – p-value calculated by Fisher’s Exact Test. Numbers in bold number reflect statistically significant associations.

Overall survival at 3 years was 47%. No statistical significance was observed on the overall survival according to gender, tumor site and tobacco and alcohol use. But, as expected, the overall survival was better for those patients with early T stage (63.0% initial vs. 36.8% advanced, p = 0.010) and negative N stage (64.9% negative vs. 34.4% positive, p = 0.001). No significant association was found between any other clinical markers and overall survival rates (Additional file 2: Table S2).

**Figure 2:**
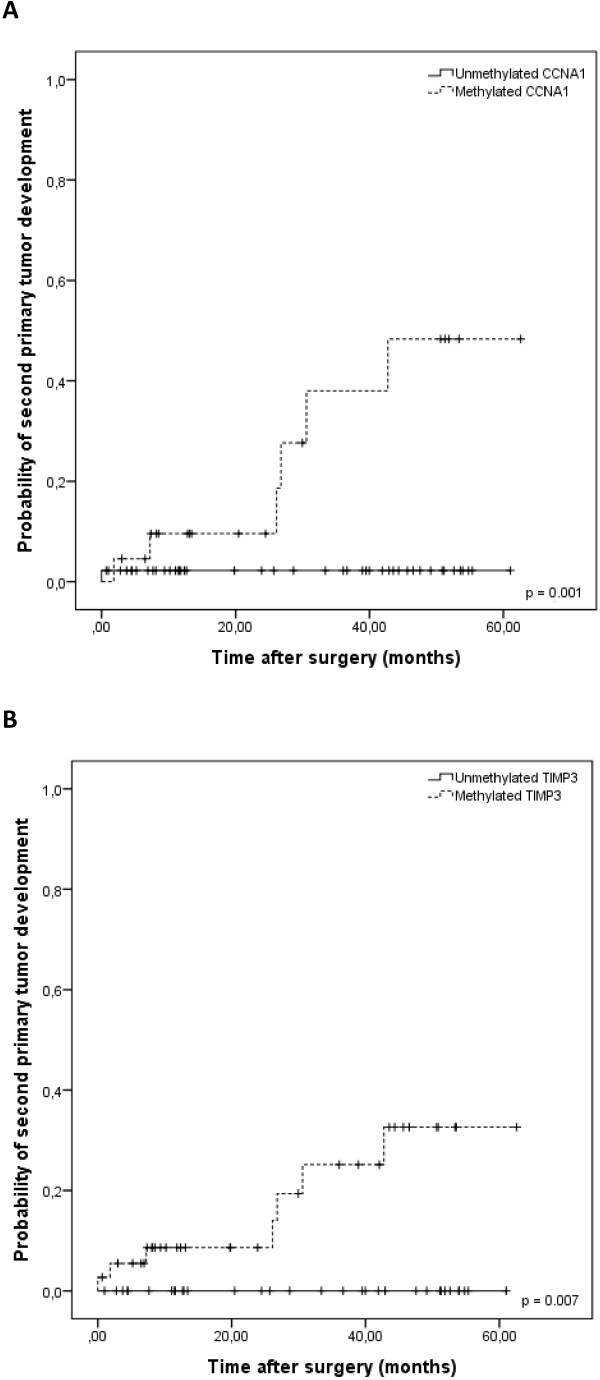
Kaplan-Meier curve comparing the probability of second primary tumor development in patients carrying methylated or unmethylated versions of CCNA1 (A) and TIMP3 (B) in HNSCC samples.

The analyses of overall survival were not able to identify any significant associations with the hypermethylation status of the five investigated genes in the HNSCC cases (Table 4), but, given the association between *CCNA1* and *TIMP3* hypermethylation and the development of SPT, the second primary tumor-free survival at 3-years was also evaluated (Table 4). Notably, HNSCC patients carrying tumors with methylated versions of *CCNA1* and *TIMP3* genes experienced an increased probability of developing SPT in comparison to patients whose tumors presented unmethylated versions of these two genes (*CCNA1*: 38.0% methylated vs. 2.2% unmethylated, p = 0.001; and *TIMP3*: 25.1% methylated vs. 0% unmethylated, p = 0.007; log-rank test; Figure 2). A significantly higher risk of developing second primary tumors was observed for patients carrying tumors with methylated *CCNA1* (HR = 13.95, 95% CI = 1.67-116.33; p = 0.015)*,* but the same was not observed for methylated *TIMP3* tumors (HR = 68.62, 95% CI = 0.15-30191.20; p = 0.173). The independent effect of *CCNA1* methylation and significant clinical features on the probability of second primary tumor development was analyzed using a Cox regression model. This multivariate analysis was not able to detect any independent factor.

**Table 4:** 3-year overall survival and SPT-free survival rates according to the methylation status of the five selected genes and the 5-gene panel

**Variables**	**Methylation status**	**Overall survival (%)**	**p**	**SPT-free survival (%)**	**p**
*CCNA1*	U	50.2	0.578	97.8	**0.001**
	M	34.5		62.0	
*DAPK*	U	46.3	0.914	94.1	0.292
	M	45.7		79.7	
*MGMT*	U	44.4	0.714	88.5	0.527
	M	48.2		77.4	
*SFRP1*	U	43.1	0.494	91.7	0.160
	M	43.9		79.1	
*TIMP3*	U	52.8	0.183	100	**0.007**
	M	40.5		74.9	
5-gene panel	U	48.2	0.176	100	0.091
	M	38.9		81.4	

SPT, Second Primary Tumor; U – unmethylated; M – methylated. Numbers in bold number reflect statistically significant associations.

## Discussion

The treatment approach and consequently the prognosis of HNSCC patients is mainly determined by the stage at presentation through the evaluation of the tumor extent, the presence of lymph-node and distant metastases and several histopathological parameters evaluated after surgery. Disappointingly, despite the evolution in patient management, the overall survival of HNSCC has not markedly improved in recent decades [34]. In HNSCC, late diagnosis and the development of loco-regional recurrences are responsible for the poor prognosis observed. Besides them, another common reason for treatment failure in HNSCC cases is the development of second primary tumors (SPT) [4]. HNSCC patients show a 10–30 times greater chance of developing SPT [35].

In order to identify new molecular markers for prognosis of HNSCC patients, we used QMSP to assess the methylation status of 19 genes in HNSCC samples collected during surgical treatment. *CCNA1*, *DAPK*, *MGMT, SFRP1* and *TIMP3* were found frequently and specifically methylated in HNSCC specimens.

A small number of studies have reported a relatively frequent hypermethylation of these genes in HNSCC [36-46]. According to them, *CCNA1* methylation could be detected in 34-53% of HNSCC cases evaluated in three studies, while *DAPK* gene methylation was detected in 21-74% of tumors examined by six studies. *MGMT* hypermethylation was detected in 22-50% of tumors examined by four independent research groups, *SFRP1* was methylated in 24-35% of tumors examined in two different studies and *TIMP3* methylation was detected in 10-72% of tumors evaluated in two studies. Consistent with this, we also found *CCNA1* (33%), *DAPK* (51%), *MGMT* (21%) and *TIMP3* (53%) frequently methylated in HNSCC samples. In contrast, we were able to detect *SFRP1* methylation in 62% of the HNSCC samples, a frequency higher than observed previously.

To our knowledge, this is the first study to show a significant association between the presence of *TIMP3* and *CCNA1* aberrant methylation in the primary HN carcinomas and the development of SPT. *Tissue inhibitor of metalloproteinases 3* (*TIMP3*) belongs to a family of genes that inhibit matrix metalloproteinases (MMPs), a group of peptidases involved in degradation of the extracellular matrix (ECM). TIMP3 exerts its anti-proteolytic function either at the invasion front of an infiltrating tumor to quench tumor-associated ECM degrading activity or in the stroma itself, where soluble proteases liberate ECM-tethered factors that assist the cancer cell in migration and invasion. Numerous studies have indicated that TIMPs inhibit cellular invasion, tumorigenesis, metastasis and angiogenesis [47]. Therefore, the hypermethylation of *TIMP3* and, consequently, its transcriptional repression would hinder its function as inhibitors of matrix metalloproteinases (MMPs), thus contributing to the degradation of the extracellular matrix. A previous study [48] reported that an increased expression of *MMP9* in the histologically negative surgical margins of HNSCC was associated with the development of SPT. *MMP9* encodes a gelatinase that degrades type IV collagen, the major constituent of basement membrane. The lateral spread of clones from malignant tumors involves the occurrence of multiple factors necessary for cell motility to penetrate the extracellular matrix [49]. Thus, the inhibition of *TIMP3* by hypermethylation and, consequently, the loss of the regulating activity of the MMP extracellular matrix degradation may contribute to the development of SPT. Sun et al. [50] showed the detection of *TIMP3* hypermethylation in salivary rinse samples collected at diagnoses associated with local recurrence-free survival in patients with HNSCC. In a recent study, our group demonstrated that the detection of *TIMP3* hypermethylation in salivary rinse collected, not only at diagnosis, but also six months after the last curative treatment is an independent prognostic factor for HNSCC patients [24].

The protein encoded by *cyclin A1* (*CCNA1*) belongs to the highly conserved cyclin family, whose members are characterized by a dramatic periodicity in protein abundance through the cell cycle. Cyclins function as regulators of CDK kinases. CCNA1 cyclin was found to bind to important cell cycle regulators, such as Rb family proteins, transcription factor E2F-1, and the p21 family proteins [51,52]. A previous study found promoter hypermethylation of the *cyclin A1* gene in 45% of primary HNSCC tissue samples evaluated, as well as in multiple cell lines. Rivera et al. [53] could show that *CCNA1* is a downstream target of *p53* and it can induce apoptosis and G2M arrest if up-regulated. We sought that loss of *CCNA1* expression though promoter hypermethylation might be involved in early oncogenic events, down regulating apoptosis and cell cycle arrest, therefore contributing to a proliferative advantage to cells in precursor lesions and giving rise to the expansion of a clonal population of progenitor cells susceptible to new oncogenic events. These lesions can accumulate oncogenic events to give rise to the development of SPT.

Although the presence of fields with a high risk of development of second primary tumors is indicated by certain clinical lesions such as erythroplakia and leukoplakia [54], most premalignant fields are not clinically detectable and others can extend well beyond the clinically visible area [55,56]. Previous studies have already supported the theory of field cancerization, which refers to the presence of malignant or premalignant changes in the entire field of apparently normal tissue adjacent to the tumor in response to a carcinogen exposition [11]. According to this theory, the development of SPT represents the progression of multiple separate genetically altered mucosal foci. However, recent studies have been reporting that at least a proportion of these SPT arise from residual portions of a single contiguous preneoplastic field after the complete resection of the index tumor. According to them, a stem cell acquires genetic alterations and forms a patch with genetically altered daughter cells. As a result of subsequent genetic alterations, the stem cell escapes normal growth control, gains growth advantage, and develops into an expanding clone. The lesion laterally displaces the normal epithelium and additional genetic hits give rise to various subclones within the field. Different clones diverge at a certain point with respect to genetic alterations but do share a common clonal origin, and as a result of the process of clonal divergence and selection, eventually a subclone evolves into invasive cancer [57]. Our results suggest that some of these genetic alterations could be the aberrant methylation of *CCNA1* and *TIMP3* genes. Along the same line, our group has also demonstrated that the overexpression of *MMP9* in histologically negative HNSCC margins was significantly correlated to a high risk of developing SPT [48].

## Conclusions

In summary, our results showed that *CCNA1*, *DAPK*, *MGMT, SFRP1* and *TIMP3* are frequently and specifically hypermethylated in HNSCC samples. In spite of the small number of samples evaluated, we demonstrated for the first time that the hypermethylation of *CCNA1* and *TIMP3* are significantly correlated to the development of SPT. Based on these results, we may speculate that the methylation pattern of these genes in HNSCC, could be a helpful marker for the identification of subjects at risk of new neoplastic evolution. Of note, the confidence intervals observed in the analyses of hazard ratios are large and this may be due to the small sample size evaluated. Despite of this, the statistically significance observed in the association through the log-rank analyses for both genes and in the Cox regression for *CCNA1* and STP denotes the potential of these markers as clinically relevant. The possibility of evaluating the primary tumor to predict the risk for the development of second primary tumors is relevant given the difficulty of identifying premalignant fields in the upper aerodigestive tract and the fact that the whole mucosa would have to be assessed, representing a very invasive diagnostic method. Further validation of these results requires studies with larger patient groups and longer follow-up period, but by achieving a good predictive negative value, this QMSP approach could constitute an alternative in predicting the risk for the development of SPT, allowing the use of preventive measures, with more frequent clinical monitoring of these patients and maybe select patients candidates for adjuvant treatment.

## Competing interests

The authors declare that they have no competing interests.

## Authors’ contributions

MMR and ACC performed the research, collected clinical data, analyzed data and wrote the paper. ALB performed some of the wet bench experiments. CZO performed the statistical analysis. ALC and LFK recruited patients and collected tissue samples. ALV designed and coordinated the study and wrote the paper. All authors have read and approved the final manuscript.

## Supplementary Material

Additional file 1: Table S1 Primers and probes used in the QMSP assays. Click here for file

Additional file 2: Table S2 SPT control and overall survival rates according to the clinical variables. Click here for file
