# Supplementary material for: TIMP3 and CCNA1 hypermethylation in HNSCC is associated with an increased incidence of second primary tumors
Source: J Transl Med. 2013 Dec 20;11:316. doi: 10.1186/1479-5876-11-316 (PMC3884019; doi:10.1186/1479-5876-11-316)
Supplement: Additional file 1: Table S1 — Primers and probes used in the QMSP assays. [file 1479-5876-11-316-S1.docx]

Supplementary Table S1 – Primers and probes used in the QMSP assays.

| **Gene** | **Forward 5´-3´** | **Probe 6FAM 5´-3´TAMRA** | **Reverse 5´-3´** |
| --- | --- | --- | --- |
| *ACTB* ^1^ | TGGTGATGGAGGAGGTTTAGTAAGT | ACCACCACCCAACACACAATAACAAACACA | AACCAATAAAACCTACTCCTCCCTTAA |
| *CALCA*^1^ | GTTTTGGAAGTATGAGGGTGACG | ATTCCGCCAATACACAACAACCAATAAACG | TTCCCGCCGCTATAAATCG |
| *CCNA1* ^2^ | TCGCGGCGAGTTTATTCG | CGTTATGGCGATGCGGTTTCGG | CCGACCGCGACAAACG |
| *CCND2* ^2^ | TTTGATTTAAGTATGCGTTAGAGTACG | TAGGGTCGATCGTGTTGGCGGCGATT | ACTTTCTCCCTAAAAACCGACTACG |
| *COX2* ^1^ | GTTTTGGAAGTATGAGGGTGACG | ATTCCGCCAATACACAACAACCAATAAACG | TTCCCGCCGCTATAAATCG |
| *CDH1* ^1^ | AATTTTAGGTTAGAGGGTTATCGCGT | CGCCCACCCGACCTCGCAT | TCCCCAAAACGAAACTAACGAC |
| *CDKN2B* ^1^ | AGGAAGGAGAGAGTGCGTCG | TTAACGACACTCTTCCCTTCTTTCCCACG | CGAATAATCCACCGTTAACCG |
| *DAPK* ^3^ | GGATAGTCGGATCGAGTTAACGTC | TTCGGTAATTCGTAGCGGTAGGGTTTGG | CCCTCCCAAACGCCGA |
| *DCC* ^2^ | TTGTTCGCGATTTTTGGTTTC | GCGCTAAACAAAAAAACTCCGAAAA | ACCGATTACTTAAAAATACGCG |
| *HIC1* ^1^ | GTTAGGCGGTTAGGGCGTC | CAACATCGTCTACCCAACACACTCTCCTACG | CAACAACTACCTAAAATAACCGAACG |
| *HIN1* ^5^ | TAGGGAAGGGGGTACGGGTTT | ACTTCCTACTACGACCGACGAACC | CGCTCACGACCGTACCCTAA |
| *MGMT* ^3^ | CGAATATACTAAAACAACCCGCG | AATCCTCGCGATACGCACCGTTTACG | GTATTTTTTCGGGAGCGAGGC |
| *MT1G* ^6^ | CGTTTAAGGGATTTTGTATTTGGTTTAT | CGCGATCCCGACCTAAACTATACGCA | CCGCTAAATCCGCACCG |
| *RARβ* ^4^ | GGGATTAGAATTTTTTTATGCGAGTTGT | TGTCGAGAACGCGAGCGATTCG | TACCCCGACGATACCCAAAC |
| *SFRP1* ^6^ | GAATTCGTTCGCGAGGGA | CCGTCACCGACGCGAAAACCAAT | AAACGAACCGCACTCGTTACC |
| *SOCS1 ^7^* | GCGTCGAGTTCGTGGGTATTT | ACAATTCCGCTAACGACTATCGCGCA | CCGAAACCATCTTCACGCTAA |
| *TGFβR2* ^6^ | CGGTGAGGGGTAGTTGAAAGTC | CGACGTCCAACCCCTAACTCTC | CAACTTCAACTCAACGCTACG |
| *THBS1* ^1^ | CGACGCACCAACCTACCG | ACGCCGCGCTCACCTCCCT | GTTTTGAGTTGGTTTTACGTTCGTT |
| *TIMP3* ^1^ | GCGTCGGAGGTTAAGGTTGTT | AACTCGCTCGCCCGCCGAA | CTCTCCAAAATTACCGTACGCG |
| *UCHL1* ^2^ | TCGGCGAGTGAGATTGTAAGGTT | GGTTCGGTCGTATTATTTCGCGTTGCGTACGG | CGATCGCGACCAAATAAATACG |

(1) Eads CA, Lord RV, Wickramasinghe K, et al. Epigenetic patterns in the progression of esophageal adenocarcinoma. *Cancer research*. 2001;61(8):3410.

(2) Carvalho AL, Jeronimo C, Kim MM, et al. Evaluation of promoter hypermethylation detection in body fluids as a screening/diagnosis tool for head and neck squamous cell carcinoma. *Clinical Cancer Research*. 2008;14(1):97-107.

(3) Harden SV, Tokumaru Y, Westra WH, et al. Gene promoter hypermethylation in tumors and lymph nodes of stage I lung cancer patients. *Clinical cancer research*. 2003;9(4):1370.

(4) Hoque, MO, E. Rosenbaum, WH Westra, et al. Quantitative assessment of promoter methylation profiles in thyroid neoplasms. *Journal of Clinical Endocrinology and Metabolism.* 2005; 90(7):4011.

(5) Fackler MJ, McVeigh M, Mehrotra J, et al. Quantitative multiplex methylation-specific PCR assay for the detection of promoter hypermethylation in multiple genes in breast cancer. *Cancer research*. 2004;64(13):4442.

(6) Weisenberger DJ, Siegmund KD, Campan M, et al. CpG island methylator phenotype underlies sporadic microsatellite instability and is tightly associated with BRAF mutation in colorectal cancer. *Nature genetics*. 2006;38(7):787-793.

(7) Muller HM, Widschwendter A, Fiegl H, et al. DNA Methylation in Serum of Breast Cancer Patients. *Cancer research*. 2003;63(22):7641.

.
