# Supplementary material for: TIMP3 and CCNA1 hypermethylation in HNSCC is associated with an increased incidence of second primary tumors
Source: J Transl Med. 2013 Dec 20;11:316. doi: 10.1186/1479-5876-11-316 (PMC3884019; doi:10.1186/1479-5876-11-316)
Supplement: Additional file 2: Table S2 — SPT control and overall survival rates according to the clinical variables. [file 1479-5876-11-316-S2.docx]

Supplementary Table S2 – SPT control and overall survival rates according to the clinical variables

| **Variables** | **Categories** | **3-year overall survival (%)** | p | **3-year SPT control (%)** | **p** |
| --- | --- | --- | --- | --- | --- |
| Gender | Male | 44.5 | 0.224 | 82.7 | 0.491 |
|  | Female | 57.1 |  | 100 |  |
|  |  |  |  |  |  |
| Tobacco consumption | No | 44.4 | 0.947 | 100 | 0.999 |
|  | Yes | 47.3 |  | 85.1 |  |
|  |  |  |  |  |  |
| Alcohol consumption | No | 46.2 | 0.852 | 100 | 0.806 |
|  | Yes | 47.2 |  | 84.5 |  |
|  |  |  |  |  |  |
| Tumor site | Oral cavity | 44.1 | 0.781 | 94.4 | 0.368 |
|  | Larynx | 54.5 |  | 86.2 |  |
|  | Oropharynx | 37.5 |  | 80 |  |
|  | Hypopharynx | 50 |  | 100 |  |
|  |  |  |  |  |  |
| T Stage | T1/T2 | 63 | **0.01** | 100 | 0.087 |
|  | T3/T4 | 36.8 |  | 70 |  |
|  |  |  |  |  |  |
| N Stage | Negative | 64.9 | **0.001** | 91.7 | 0.226 |
|  | Positive | 34.4 |  | 88.1 |  |
|  |  |  |  |  |  |
| Vascular embolization | Negative | 50.8 | 0.2 | 88.9 | 0.36 |
|  | Positive | 41.7 |  | 81.5 |  |
|  |  |  |  |  |  |
| Perineural invasion | Negative | 53.5 | 0.337 | 89.6 | 0.764 |
|  | Positive | 38 |  | 78.9 |  |
|  |  |  |  |  |  |
| Margins | Negative | 53.1 | 0.299 | 86 | 0.194 |
|  | Positive | 25 |  | 85.7 |  |
|  |  |  |  |  |  |
| Lymph node involvement | Negative | 60.5 | 0.198 | 92.3 | 0.149 |
|  | Positive | 43.3 |  | 82.3 |  |
|  |  |  |  |  |  |
| Curative treatment | S | 52.7 | **0.041** | 100 | 0.311 |
|  | R | 66.7 |  | 100 |  |
|  | S + R | 48.7 |  | 87.9 |  |
|  | S + R + C | 66.7 |  | 88.9 |  |
|  | R + C | 25 |  | 66.7 |  |
|  |  |  |  |  |  |

SPT, Second Primary Tumor; S, surgery; R, radiotherapy; C, chemotherapy
